# We Should Pay More Attention to Sex Differences to Predict the Risk of Severe COVID-19: Men Have the Same Risk of Worse Prognosis as Women More Than 10 Years Older

**DOI:** 10.2188/jea.JE20220056

**Published:** 2023-01-05

**Authors:** Yumi Matsushita, Tetsuji Yokoyama, Kayoko Hayakawa, Nobuaki Matsunaga, Hiroshi Ohtsu, Sho Saito, Mari Terada, Setsuko Suzuki, Shinichiro Morioka, Satoshi Kutsuna, Shinya Tsuzuki, Hisao Hara, Akio Kimura, Norio Ohmagari

**Affiliations:** 1 Department of Clinical Research, National Center for Global Health and Medicine, Tokyo, Japan; 2 Department of Health Promotion, National Institute of Public Health, Saitama, Japan; 3 AMR Clinical Reference Center, National Center for Global Health and Medicine, Tokyo, Japan; 4 Disease Control and Prevention Center, National Center for Global Health and Medicine, Tokyo, Japan; 5 Center for Clinical Sciences, National Center for Global Health and Medicine, Tokyo, Japan; 6 Faculty of Medicine and Health Sciences, University of Antwerp, Antwerpen, Belgium; 7 Department of Cardiology, National Center for Global Health and Medicine, Tokyo, Japan; 8 Department of Emergency Medicine and Intensive Care Unit Center Hospital of the National Center for Global Health and Medicine, Tokyo, Japan

**Keywords:** COVID-19, infectious disease, sex, priority, age

## Abstract

**Background:**

Prioritization for novel coronavirus disease 2019 (COVID-19)-related health policies usually considers age and certain other characteristics, but sex is rarely included, despite the higher risk of severe disease in men. The aim of this study was to compare the impact of sex and age on the severity of COVID-19 by estimating the age difference in years for which the risk for men versus women is the same.

**Methods:**

We analyzed 23,414 Japanese COVID-19 inpatients aged 20–89 years (13,360 men and 10,054 women). We graded the severity of COVID-19 (0 to 5) according to the most intensive treatment required during hospitalization. The risk of grade 2/3/4/5 (non-invasive positive pressure ventilation/invasive mechanical ventilation/extracorporeal membrane oxygenation/death), grade 3/4/5, and separately grade 5 was analyzed using a multiple logistic regression model.

**Results:**

The odds ratio (OR) of grades 2/3/4/5, 3/4/5 (primary outcome), and 5 for men relative to women was 2.76 (95% CI, 2.44–3.12), 2.78 (95% CI, 2.42–3.19), and 2.60 (95% CI, 2.23–3.03), respectively, after adjustment for age and date of admission. These risks for men were equivalent to those for women 14.1 (95% CI, 12.3–15.8), 11.2 (95% CI, 9.7–12.8), and 7.5 (95% CI, 6.3–8.7) years older, respectively.

**Conclusion:**

The risks of worse COVID-19 prognosis (grades 3/4/5) in men were equivalent to those of women 11.2 years older. Reanalyzing data extracted from four previous studies also revealed a large impact of sex difference on the severity of COVID-19. We should pay more attention to sex differences to predict the risk of COVID-19 severity and to formulate public health policy accordingly.

## INTRODUCTION

Studies have begun to unravel the correlation between age and COVID-19 severity and mortality. It is clear that older patients are more likely to suffer from progression, severe disease, and death from COVID-19 than younger patients. This is reflected in the oft-reported finding that the mean age of patients with severe COVID-19 disease is higher than of those with milder disease,^1^^–^^4^ and that the mean age of survivors is lower than of those who succumb.^1^^,^^5^^,^^6^ Furthermore, the mean age of patients with progressive disease is higher than of those with stable disease,^1^^–^^7^ and the proportion of patients with severe disease increases with age.^1^^–^^4^

This state of affairs is reflected in the policies adopted by many countries to prioritize the older members of the population regarding preventive and therapeutic measures, such as vaccination priorities and drug treatments. Of course, these policies must also take many other factors into account; for example, medical workers, essential workers, and others prioritized for vaccination. Many studies have confirmed a correlation between sex and COVID-19 susceptibility, whereby men have a higher risk of severe disease than women.^1^ Some reports suggest that the difference between men and women may be due to hormonal status,^8^^–^^10^ but the mechanism responsible for this sex difference has not been clarified. Sex has not been considered for the prioritization of preventive and therapeutic policies in most countries.^11^^,^^12^ This may result from minimal investigations evaluating quantitative differences for the impact of age and sex on severity of COVID-19. Here, we analyzed the impact of sex on severity of COVID-19 compared to the impact of age using a large-scale COVID-19 registry containing data on hospitalized COVID-19 patients in Japan (COVIREGI-JP).

## METHODS

### Study design

This was a study using data from COVIREGI-JP, a large-scale registry of hospitalized COVID-19 patients in Japan, that contains data on patients’ characteristics and clinical course during hospitalization. Prior to the beginning of this work, we disseminated information about the registry on the website of the National Center for Global Health and Medicine (the primary investigator’s affiliation), webinar for journalists, press release, and other outlets and asked hospitals to participate in COVIREGI-JP and register the patients’ information voluntarily. A total of 576 hospitals were involved with this registry system. The details have been reported elsewhere.^13^^–^^16^ Criteria for enrollment in this registry were a positive SARS-CoV-2 test^13^ and inpatient treatment. For patients with a history of ≥2 COVID-19 hospitalizations, each was entered separately. Nonetheless, such individuals were omitted from the present study, as it was not possible to link the different hospitalization data on one specific patient. This meant the most intensive treatment regimen (ie, the outcome for the current analysis) was not identifiable. In addition, age was restricted to 20–89 years, and in order to prevent any confounding effects of ethnicity, non-Japanese patients were not included.

### Dataset

Data items entered by May 20, 2021 were extracted as follows: sex and age, outcome at the time of discharge (death or alive/hospital transfer), and nature of supportive care while in hospital (shown in the section “Definition of disease grade” below). Comorbidities at admission were recorded as follows: a history of myocardial infarction, congestive heart failure, peripheral vascular disease, cerebrovascular disorders, hemiplegia, dementia, chronic obstructive pulmonary disorder (COPD), chronic lung diseases other than COPD, bronchial asthma, mild liver disease, mild to severe liver dysfunction, peptic ulcer, hypertension, hyperlipidemia, diabetes mellitus, obesity, moderate to severe renal dysfunction, maintenance hemodialysis before hospitalization, solid cancers, leukemia, lymphoma, metastatic solid cancers, connective tissue disease, and human immunodeficiency virus infection. Four categories of smoking history were noted: current (smoking until just prior to COVID-19), former, never smoker, and unknown.

### Patients

The COVIREGI-JP comprised 38,059 patients hospitalized from January 26, 2020 to April 27, 2021. All were either discharged or deceased by May 20, 2021. Patients omitted from the study for the above reasons were excluded in the following order: non-Japanese (*n* = 4,419), sex unknown (*n* = 19), outside the age range (*n* = 3,093), transferred from another hospital (*n* = 3,600), transferred to another hospital (*n* = 2,484), still hospitalized or outcome unknown (*n* = 736), treatment procedures unknown (*n* = 201), and admission and/or discharge dates lacking (*n* = 93). Thus, 23,414 patients aged 20–89 years (13,360 men and 10,054 women) remained for this analysis (Table 1).

**Table 1.** Definition of grade according to the most intensive treatment or death and the frequency of each severity grade by sex and age groups among 23,414 COVID-19 patients registered in Japan

Age, years	*N*	Grade					

		0. No oxygen	1. nasal cannula/ oxygen masks	2. high-flow oxygen devices/NIPPV	3. invasive mechanical ventilation	4. ECMO	5. death (regardless of treatment)
Men							
20–29	1,666	97.1%	2.6%	0.1%	0.2%	0.0%	0.0%
30–39	1,595	89.0%	9.6%	1.0%	0.4%	0.1%	0.0%
40–49	2,157	78.3%	18.5%	1.9%	0.9%	0.2%	0.2%
50–59	2,593	67.6%	27.2%	2.2%	2.0%	0.3%	0.7%
60–69	2,114	55.0%	35.1%	3.6%	2.7%	0.1%	3.5%
70–79	1,997	47.1%	37.5%	3.5%	2.5%	0.2%	9.3%
80–89	1,238	38.7%	33.0%	1.9%	0.2%	0.0%	26.2%
Total	13,360	67.8%	23.9%	2.1%	1.4%	0.1%	4.5%
Women							
20–29	1,546	98.3%	1.5%	0.1%	0.1%	0.0%	0.0%
30–39	1,080	96.2%	3.6%	0.1%	0.1%	0.0%	0.0%
40–49	1,180	90.8%	8.0%	0.4%	0.2%	0.1%	0.5%
50–59	1,490	82.3%	16.3%	0.7%	0.3%	0.0%	0.3%
60–69	1,401	73.2%	23.6%	1.3%	1.1%	0.0%	0.9%
70–79	1,749	63.9%	28.9%	2.1%	0.5%	0.0%	4.5%
80–89	1,608	52.2%	34.3%	1.3%	0.2%	0.0%	12.0%
Total	10,054	78.0%	17.8%	0.9%	0.4%	0.0%	2.9%

Percentage values are for row.

### Definition of disease grade

The severity of COVID-19 was graded according to the most intensive treatment received in hospital, or death, as shown in Table 1, using the following stratification: grade zero (no oxygen required; ie, patients never received any supplemental oxygen); grade 1 (patients were supported with noninvasive mechanical ventilation or supplemental oxygen); grade 2 (patients supported with high-flow oxygen or non-invasive positive pressure ventilation [NIPPV]); grade 3 (patients required invasive mechanical ventilation [IMV]); grade 4 (extracorporeal membrane oxygenation [ECMO]); grade 5 (death in hospital no matter which treatment was given). We analyzed grades 2/3/4/5 together in one pool, grades 3/4/5 as the primary outcome, and, finally, grade 5 separately.

### Statistical analysis

The frequencies of COVID-19 grades are shown as percentages with 95% confidence intervals (CIs). Continuous variables were summarized as mean, median, and quartiles. The odds ratio (OR) and 95% CI of grades 3/4/5 according to sex and age were calculated using a multiple logistic regression model adjusted for potential confounding variables, including date of admission, smoking status, body mass index (BMI), and comorbidities (see Table 2, footnote), where the date of admission was categorized into seven periods with 2-month intervals and used for the adjustment. As covariates, we used smoking status, BMI, and comorbidities, which are known risk factors for COVID-19.^16^^,^^17^ We adjusted for comorbidities that were significantly associated with COVID-19 severity in our previous study.^16^ Hospital capacity was not examined, so date of admission was added as an adjustment factor. To estimate how many years difference between men and women was necessary to equalize the risk of COVID-19 severity, the following multiple logistic regression model was used:

$$\begin{array}{rl} logit(p) & =\alpha +\beta_{1}\times \text{sex}+\beta_{2}\times \text{age}\\ & \;+\sum_{i}(\beta_{i}\times {\text{confounding variable}}_{i}) \end{array}$$

where *p* is the probability of having grades 3/4/5, *α* is the intercept, *β*_1_, *β*_2_, and *β_i_* are partial regression coefficients, sex is coded as 0 (women) or 1 (men), and age is expressed in years as a continuous variable. In this model, the OR of aging by Y years is exp(*β*_2_ × Y); that of men vs women is exp(*β*_1_). Thus, the OR of aging by *β*_1_/*β*_2_ years is equivalent to that of men vs women because exp(*β*_2_ × (*β*_1_/*β*_2_)) = exp(*β*_1_). The confidence interval for *β*_1_/*β*_2_ was calculated by the delta method using NLEST macro.^18^ The same analysis was repeated for grades 2/3/4/5 and grade 5. In the same manner, we also estimated the ratio of *β*_1_/*β*_2_ in previous studies that reported ORs or hazard ratios (HRs) for both sex and age to assess the risk COVID-19^17^^,^^19^^–^^21^ (see eMaterial 1). The statistical significance level was set at 5% in two-sided tests. ORs not containing 1 within the 95% CI were considered significant. All statistical analyses were conducted using SAS version 9.4 (SAS Institute Inc., Cary, NC, USA).

**Table 2.** Odds ratios of severe grades according to sex and age in 23,414 COVID-19 patients registered in Japan

Adjusted variables	Grade 2/3/4/5 (HF,NIPPV/IMV/ECMO/death), *n* = 1,531						Grade 3/4/5 (IMV/ECMO/death), *n* = 1,149						Grade 5 (death), *n* = 902					
		
	Age		Men, vs women				Age		Men, vs women				Age		Men, vs women			
					
	β_2_	OR (95% CI) per +10 years^a^	β_1_	OR (95% CI)	Equivalent risk in age, years: β_1_/β_2_ (95% CI)		β_2_	OR (95% CI) per +10 years	β_1_	OR (95% CI)	Equivalent risk in age, years: β_1_/β_2_ (95% CI)		β_2_	OR (95% CI) per +10 years	β_1_	OR (95% CI)	Equivalent risk in age, years: β_1_/β_2_ (95% CI)	
Sex/age	0.069	2.00 (1.92–2.08)	1.021	2.78 (2.46–3.13)	14.7	(13.0–16.5)	0.087	2.38 (2.25–2.50)	1.026	2.79 (2.43–3.20)	11.9	(10.2–13.5)	0.123	3.42 (3.17–3.70)	0.961	2.62 (2.25–3.04)	7.8	(6.6–9.1)
Sex/age and date of admission^b^	0.072	2.06 (1.97–2.14)	1.015	2.76 (2.44–3.12)	14.1	(12.3–15.8)	0.091	2.48 (2.35–2.62)	1.022	2.78 (2.42–3.19)	11.2	(9.7–12.8)	0.128	3.58 (3.30–3.88)	0.956	2.60 (2.23–3.03)	7.5	(6.3–8.7)
Sex/age, date of admission^b^, and smoking	0.070	2.02 (1.94–2.11)	0.916	2.50 (2.19–2.85)	13.0	(11.1–14.9)	0.089	2.43 (2.29–2.57)	0.940	2.56 (2.20–2.97)	10.6	(8.9–12.3)	0.125	3.49 (3.21–3.78)	0.865	2.38 (2.01–2.81)	6.9	(5.6–8.3)
Sex/age, date of admission^b^, and body mass index	0.074	2.10 (2.01–2.20)	1.012	2.75 (2.43–3.11)	13.6	(11.9–15.3)	0.091	2.48 (2.35–2.63)	1.040	2.83 (2.46–3.26)	11.4	(9.8–13.0)	0.126	3.51 (3.23–3.81)	0.985	2.68 (2.29–3.13)	7.8	(6.6–9.1)
Sex/age, date of admission^b^, and comorbidities^c^	0.064	1.90 (1.81–1.99)	0.914	2.49 (2.19–2.83)	14.2	(12.1–16.3)	0.080	2.23 (2.10–2.37)	0.915	2.50 (2.15–2.89)	11.4	(9.5–13.3)	0.117	3.22 (2.94–3.53)	0.824	2.28 (1.93–2.69)	7.0	(5.6–8.5)
All^c^	0.064	1.90 (1.81–2.00)	0.843	2.32 (2.02–2.66)	13.1	(10.9–15.3)	0.079	2.21 (2.07–2.35)	0.872	2.39 (2.04–2.80)	11.0	(8.9–13.1)	0.115	3.16 (2.88–3.47)	0.789	2.20 (1.84–2.63)	6.8	(5.3–8.4)

CI, confidence interval; ECMO, extracorporeal membrane oxygenation; HF, high-flow oxygen devices; IMV, invasive mechanical ventilation; NIPPV, non-invasive positive pressure ventilation; OR, odds ratio.

β_1_, partial regression coefficient of men vs women; β_2_, partial regression coefficient of age in years. Partial regression coefficients, ORs, and CIs were estimated using a multiple logistic regression model.

^a^OR per +10 years increase in age was calculated as exp(β_2_ × 10).

^b^The date of admission was categorized into 7 periods with 2-month intervals and used for the adjustment.

^c^Adjusted for the presense of congestive heart failure, peripheral vascular disease, cerebrovascular disorders, hemiplegia, dementia, COPD, chronic lung diseases other than COPD, mild liver disease, diabetes mellitus, obesity, moderate to severe renal dysfunction, maintenance hemodialysis before hospitalization, solid cancers, leukemia, lymphoma, metastatic solid cancers, and connective tissue disease.

## RESULTS

Including the day of admission, the mean length of the stay in hospital was 13.4 days (range, 1–307). Data regarding frequencies of disease grade by sex and age are summarized in Table 1. Grade 0 (no oxygen) was the most common, encompassing 67.8% of male patients and 78.0% of females. The most severe grades 3 and 4 were less common in patients ≥70 years of age than in those aged 60–69 years, while fatalities were very high, especially in those age 80–89 years (26.2% of men, 12.0% of women). The median of age among all patients analyzed was 54 (interquartile range [IQR], 40–69) in men and 57 (IQR, 38–74) years in women, and that of BMI was 24.3 (IQR, 22.0–27.1) kg/m^2^ and 22.0 (IQR, 19.8–25.0) kg/m^2^ for men and women, respectively. Smoking assessed as current, former, never, or unknown was recorded for 22.0%, 30.2%, 34.1%, and 13.7%, respectively, of men and 9.8%, 10.2%, 63.4%, and 16.7% of women. Hypertension was common (29.0% of men and 25.9% of women), as well as hyperlipidemia (14.2% in both sexes) and diabetes (18.0% of men and 10.9% of women), with obesity being less common (7.0% of men and 3.9% of women) as comorbidities.

**Figure 1.**
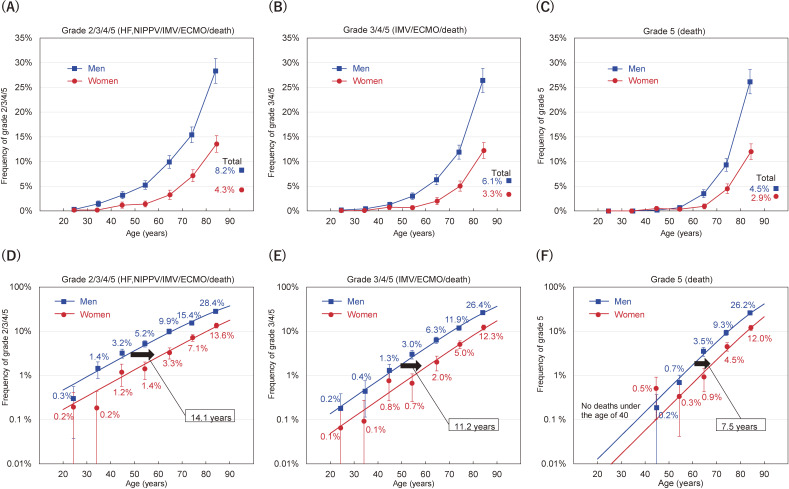
Frequency of severe disease grades according to sex and age groups among COVID-19 patients registered in Japan. The frequency with 95% confidence interval is plotted against the mean age of each group (**A**, **B**, and **C**). In the lower panel, the same data are plotted on a logarithmic Y-axis scale; the fitted lines were estimated by logistic regression models (**D**, **E**, and **F**). The estimated risks of severity grades 2/3/4/5, 3/4/5 and 5 for men were equivalent to those of women 14.1, 11.2, and 7.5 years older, respectively, after adjustment for the date of hospital admission. ECMO, extracorporeal membrane oxygenation; HF, high-flow oxygen devices; IMV, invasive mechanical ventilation; NIPPV, non-invasive positive pressure ventilation.

The frequency of severity grades or death stratified according to sex and age is shown in Figure 1A, Figure 1B, and Figure 1C. For any severity grade group, frequencies increased very rapidly with age, especially in men, reaching 26.4% with grade 3/4/5 among male patients aged 80–89 years, and 12.3% of women. Trends of increasing severity in both men and women were almost linear and parallel when the Y-axis was logarithmic (ie, exponentially), as shown in Figure 1D, Figure 1E, and Figure 1F.

The ORs of severity grades 2/3/4/5, 3/4/5, and 5 for an increment of 10 years of age were 2.06, 2.48, and 3.58, exhibiting a stronger relationship at more severe grades. In contrast, the ORs for men versus women were 2.76, 2.78, and 2.60, a similar relationship for any grade group (Table 2, 2nd row). It should be noted that the risks for men versus women were equivalent to increasing patient age by 14.1, 11.2, and 7.5 years for grades 2/3/4/5, 3/4/5, and 5, respectively, suggesting a large impact of the sex difference on the severity of COVID-19 (Table 2, 2nd row; Figure 1D, Figure 1E, and Figure 1F).

**Figure 2.**
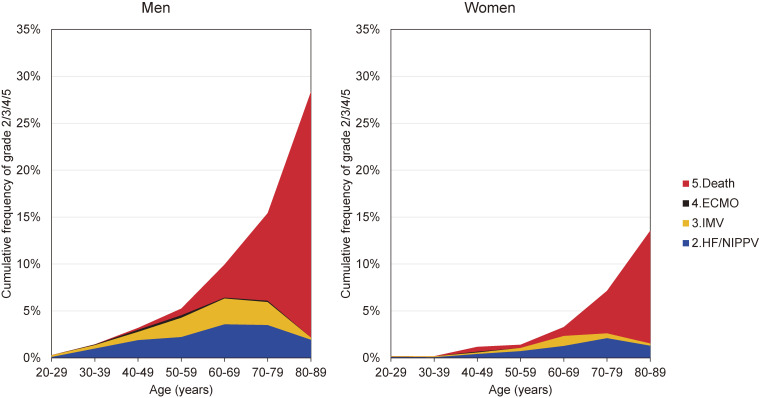
Cumulative frequency of occurrence of each severity grade or death according to sex and age among COVID-19 patients registered in Japan. ECMO, extracorporeal membrane oxygenation; HF, high-flow oxygen devices; IMV, invasive mechanical ventilation; NIPPV, non-invasive positive pressure ventilation.

The cumulative frequency of occurrence of each severity grade or death according to sex and age is shown in Figure 2. While the occurrence of grade 5 (death) markedly increased according to age, that of severe grade 3 oppositely decreased in the elderly aged 70 or over and that of moderate grade 2 also decreased in the very old patients aged 80–89 years. This contradictory occurrence of grades 2/3 and 5 in the elderly suggested that even a moderate worsening of the disease led to a sudden worsening of symptoms and death in the very old patients of COVID-19.

To explore the reasons for the higher risk of severe disease in men than women, ORs adjusted for potential confounding factors, including date of admission, smoking status, BMI, and comorbidities, were calculated (Table 2, 3rd to 6th rows). Adjusting for smoking or comorbidities slightly weakened the ORs for men versus women and shortened the years of equivalent risk in age. However, the confounding effects of these adjusted variables were very limited.

**Figure 3.**
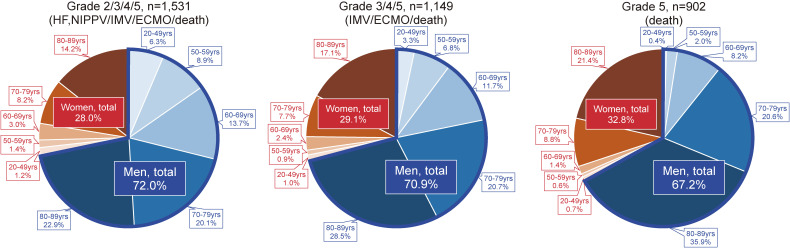
Proportion of sex and age groups among moderate to severe disease grades or death in COVID-19 patients registered in Japan. ECMO, extracorporeal membrane oxygenation; HF, high-flow oxygen devices; IMV, invasive mechanical ventilation; NIPPV, non-invasive positive pressure ventilation.

To further examine the impact of this sex difference, the proportions of sex and age groups among each severity grade in COVID-19 patients are shown in Figure 3. Two-thirds of patients suffering from grades 2/3/4/5, 3/4/5, and 5 were men (72.0%, 70.9%, and 67.2%, respectively). Especially in older adults ≥70 years of age, these values were 43.0%, 49.2%, 56.5% for men and 22.4%, 24.8%, 30.2% for women.

## DISCUSSION

We examined the associations between sex, age, and the risk of worse COVID-19 considering the confounding effects of date of admission, smoking, BMI, and comorbidities. As previous studies consistently reported, our study also confirmed an increased risk of worse COVID-19 prognosis at older age and in men. Notably, the ORs of grade 2/3/4/5, 3/4/5, and 5 for men were equivalent to those of women 14.1, 11.2, and 7.5 years older, respectively. Older age is one of the factors determining the priority of treatment and preventive measures against COVID-19 in Japan, as well as other countries.^22^ In these prioritizations, for example, age and comorbidities are considered for treatment, and age, comorbidities, and occupation (eg, medical workers, essential workers) are considered for vaccination. However, little attention is paid to the different risks in men and women. From the point of view of an individual person’s risk, the OR of severe COVID-19 for sex is quite large, being almost equivalent to that of age by 11.2 years for grades 3/4/5. For example, the risk for a 50-year-old man is the same as that of a 61.2-year-old woman. Furthermore, more than two-thirds of grade 2/3/4/5, 3/4/5, and 5 patients are men. Therefore, more attention should be paid to sex differences for considering prioritization of public health policies, such as prevention and therapeutic measures.

In the same manner as in the present study, we also estimated by how many years the risk for men was greater than for women in previous studies that reported adjusted ORs or HRs of COVID-19-related outcomes for sex and age.^23^ In a very large cohort study in England,^19^ the risk of COVID-19-related death for men was equivalent to that of women 4.9 years older (see eMaterial 1). In another community-based cohort study in England,^20^ the risk of hospitalization for men was equivalent to 22.5 years of aging in women. A prospective cohort study in New York analyzed the risk of hospital admission and for critical illness^17^; the risk for men was equivalent to that of women 17.3 and 15.7 years older, respectively. In a retrospective cohort study in Louisiana,^21^ these values for the risk of hospitalization and in-hospital death for men were 14.0 and 13.5 years, respectively. All these findings emphasize the importance of considering sex differences in formulating COVID-19-related policies.

It is not fully understood why this sex difference is so strong, but many comorbidities are prevalent in men, and the proportion of smokers is much higher in men than in women in Japan.^24^ However, these two major risk factors explain only a part of the sex difference in the risk of COVID-19 severity. This implies that there are other unidentified factors involved in mediating the effects of sex differences in COVID-19. For example, the effect of health behaviors, sex hormone-mediated immune factors, and differential expression of angiotensin-converting enzyme 2 may all contribute to explaining the different prognosis of COVID-19 in men and women.^25^ Apart from COVID-19, men are at higher risk of serious illness from many diseases; thus, for example, men have a higher mortality rate for pneumonia, influenza, and cardiovascular disease.^26^ Female life expectancy has been higher than for men for all years since 1950 in most countries.^27^ However, the reasons for the worse prognosis in men are not well understood. Although estrogen has been reported to be protective against COVID-19 and reduce severity in women, with testosterone doing the same in men,^8^^–^^10^ our data showed both men and women had a parallel increased risk of COVID-19 severity with increasing age. Our study did not yield the results expected from previous studies. This suggests that the effects of estrogen and testosterone may not be sufficient to explain the differences in severity of COVID-19 between men and women.

There are several limitations of the current study. First, we had to exclude patients with a history of ≥2 COVID-19 hospitalizations because the most intensive treatment was not identifiable and we needed to avoid the inclusion of duplicate data. Second, although we adjusted the date of admission, smoking, BMI, and comorbidities for the analysis using the multiple logistic regression model, we did not consider the interaction terms of sex and age with these covariates. In other words, we did not consider the potential heterogeneity of *β*_1_/*β*_2_ according to these background factors. Finally, the definition of outcomes (worse prognosis), population, statistical models, and adjusted covariates were different among the reanalyzed four previous studies^17^^,^^19^^–^^21^ so the results cannot be compared exactly. The heterogeneity of years in age of which risk is equivalent to male sex among the studies may be partly due to such differences. Our study has strengths, primarily being that it is first to use large-scale data of 13,360 men and 10,054 women.

### Conclusion

In conclusion, the risks of worse COVID-19 prognosis (grade 3/4/5) in men were equivalent to those of women 11.2 years older. Despite the potentially large impact of this sex difference, this has rarely been considered for the prioritization of public health policies, such as treatment and preventive measures. It is important to inform the public that men are at higher risk of worse COVID-19 prognosis than women and to more strongly emphasize that men take more extensive measures to prevent infection (eg, vaccination, hand washing, gargling, and avoiding crowding). We should pay more attention to sex as one of the factors that predicts the risk of COVID-19 severity and formulate a public health policy that reflects sex differences.

## SUPPLEMENTARY MATERIAL

eMaterial 1. Supplemental methods
