# Supplementary material for: We Should Pay More Attention to Sex Differences to Predict the Risk of Severe COVID-19: Men Have the Same Risk of Worse Prognosis as Women More Than 10 Years Older
Source: J Epidemiol. 2023 Jan 5;33(1):38–44. doi: 10.2188/jea.JE20220056 (PMC9727213; doi:10.2188/jea.JE20220056)
Supplement: Supplementary file 1 [file je-33-038-s001.pdf]

eMaterial 1. Supplemental methods

In a multiple logistic regression model or a multivariate Cox proportional hazards model in which linear predictor includes β_1×sex (men=1, women=0)+β_2×age (in years), the odds ratio (OR) or hazard ratio (HR) for men vs. women is equivalent to that for age by β_1/β_2 years because $\beta_1 \times 1 = \beta_2 \times (\beta_1/\beta_2)$. We extracted the estimates $\widehat{\beta_1}$ and $\widehat{\beta_2}$ from each paper[1-4] as follows and estimated β_1/β_2 by $\widehat{\beta_1}/\widehat{\beta_2}$. We could not calculate the confidence interval for $\widehat{\beta_1}/\widehat{\beta_2}$ because the covariance of $\widehat{\beta_1}$ and $\widehat{\beta_2}$ could not be obtained from each of the papers.

1) Lassale C, et al.[1] reported adjusted ORs for men vs. women, age per +1-year, and other variables using a multiple logistic regression model. $\widehat{\beta_1}$ and $\widehat{\beta_2}$ were obtained by the logarithms of the ORs for men vs. women and age per +1-year, respectively.

2) Price-Haywood EG, et al.[2] reported adjusted HRs for women vs. men, age per +5-years, and race using a multivariate Cox proportional hazards model. $\widehat{\beta_1}$ was obtained by the logarithm of the reciprocal of the HR for women vs. men. $\widehat{\beta_2}$ was obtained by the logarithm of the fifth root of the HR for age per +5-years. This was because the estimated HR for age per +5-years, $\widehat{\mathrm{HR}}_5$, by the Cox proportional hazards model was calculated as $\widehat{\mathrm{HR}}_5$=$e^{\widehat{\beta_2}\times 5}$ and thus $\widehat{\beta_2}$ =log($\widehat{\mathrm{HR}}_5$)/5= log($\widehat{\mathrm{HR}}_5{}^{1/5}$).

3) Petrilli CM, et al.[3] reported ORs for men vs. women, age categories of 19-44 (reference), 45-54, 55-64, 65-74, 75+ years, and other variables using a multiple logistic regression model. $\widehat{\beta_1}$ was obtained by the logarithm of the OR for men vs. women. $\widehat{\beta_2}$ was estimated by using the method

proposed by Greenland and Longnecker (5) that enables the trend estimation from summarized dose-response data, where we assigned the midpoint of the upper and the lower cut-off for each age category (i.e., 32, 50, 60, 70 years, respectively) and 80 years for the oldest group.

4) Williamson EJ, et al.[4] reported adjusted HRs for men vs. women, age categories of 18–39, 40–49, 50–59 (reference), 60–69, 70–79, 80+ years, and other variables using a multivariate Cox proportional hazards model. $\widehat{\beta_1}$ was obtained by the logarithm of the HR for men vs. women. Unfortunately, the method of Greenland and Longnecker is not applicable when the lowest or the highest category is not the reference. We plotted the logarithms of HRs for the age categories against age (assigned 29, 45, 55, 65, 75, 85 years, respectively) and found that the logarithms of the HRs increased almost exactly in a linear manner according to age (R^2=0.997). Therefore, we estimated $\widehat{\beta_2}$ by a linear regression model that regressed the logarithms of the HRs on the assigned age values.

REFERENCES

1. Lassale C, Gaye B, Hamer M, Gale CR, Batty GD. Ethnic disparities in hospitalization for COVID-19 in England: The role of socioeconomic factors, mental health, and inflammatory and pro-inflammatory factors in a community-based cohort study. *Brain Behav Immun.* 2020;88:44–9.

2. Price-Haywood EG, Burton J, Fort D, Seoane L. Hospitalization and Mortality among Black Patients and White Patients with Covid-19. *N Engl J Med.* 2020;382: 2534–43.

3. Petrilli CM, Jones SA, Yang J, Rajagopalan H, O'Donnell L, Chernyak Y, et al.. Factors associated

with hospital admission and critical illness among 5279 people with coronavirus disease 2019 in New York City: prospective cohort study. *BMJ*. 2020;369: m1966.

4. Williamson EJ, Walker AJ, Bhaskaran K, Bacon S, Bates C, Morton CE, et al. Factors associated with COVID-19-related death using OpenSAFELY. *Nature.* 2020;584:430–6.

5. Greenland S, Longnecker MP. Methods for trend estimation from summarized dose-response data, with applications to meta-analysis. *Am J Epidemiol.* 1992;135:1301-9.
